# Tuned Magnetic Properties of L1_0_-MnGa/Co(001) Films by Epitaxial Strain

**DOI:** 10.1038/srep19508

**Published:** 2016-01-19

**Authors:** Dongyoo Kim, Levente Vitos

**Affiliations:** 1 Applied Materials Physics,Department of Materials Science and Engineering,Royal Institute of Technology, Stockholm SE-100 44, Sweden; 2 Department of Physics and Materials Science, Division for Materials Theory, Uppsala University, SE-75120 Uppsala, Sweden; 3 Research Institute for Solid State Physics and Optics, Wigner Research Center for Physics, H-1525 Budapest, P.O. Box 49, Hungary

## Abstract

We demonstrate that the interface structure has a significant influence on the magnetic state of MnGa/Co films consisting of L1_0_-MnGa on face-centered-cubic Co(001) surface. We reveal an antiferromagnetic to ferromagnetic magnetization reversal as a function of the lateral lattice constant. The magnetization reversal mainly originates from localized states and weak hybridization at interface due to charge redistribution between muffin-tin spheres and interstitial region. The magnetic anisotropy energy of Mn/Co interface system is enhanced with increasing in-plane lattice constant, which is ascribed to the interface interactions and the above magnetization reversal.

Magnetic exchange coupling between ferromagnetic (FM) layers, which are connected with an interface, is one of the most important issues in spintronics devices. Nanoscale fabrication techniques enable various kinds of low dimensional magnetic structures showing unique physical properties, not found in bulk systems due to the drastically altered electronic structure and spin degrees of freedom. In particular, tailoring magnetic exchange coupling between two FM films with large perpendicular magnetic anisotropy (PMA) has been a big challenge and widely investigated due to their fundamental properties for spintronics devices and permanent magnets. It is widely accepted that the magnetic coupling between PMA alloy and 3d metal insertion layers has significant effect on magnetoresistance (MR) ratio in magnetic tunnel junction (MJT)^1^,^2^,^3^,^4^,^5^,^6^. Recently, an enhancement of MR was obtained from the core structure with insertion of 3d metals between MgO barrier and FM electrode layers in MJTs compared to non-insertion core structure^1^.

The effect of interfacial magnetic coupling is concerned as an important factor for the enhanced MR ratio. The magnetic interaction between L1_0_-MnGa alloy and 3d metal alloys has been tunned by changing the atomic compositions of the alloy^2^,^3^. Antiferromagnetic (AFM) coupling was observed in MnGa/Co bilayer film structure, but MnGa/Fe film shows FM coupling^2^. A magnetic interaction change at the interface from FM to AFM L1_0_-MnGa/Fe_1−*x*_Co_*x*_ epitaxial bilayer film was realized around 25% Co content^3^. FM interfacial coupling increases the MR ratio, whereas the AFM coupling suppresses it^1^,^2^,^3^. The magnetic coupling of the films can be adjusted by various methods, such as external magnetic and electric field, carrier doping, interface structure, and thickness of spacer layers^2^,^3^,^7^,^8^,^9^,^10^,^11^,^12^,^13^,^14^,^15^,^16^. Understanding and describing the magnetic interaction of the layered structures that are composed of more than two elements is a great challenge. The hybridization between two magnetic layers via the localized “*d*” states has been used to provide simple explanation^17^,^18^.

Materials with strong PMA have advantages compared to in-plane magnetized metals, such as smaller switching current^19^,^20^,^21^ and high magnetic anisotropy energy (MAE) promising thermal stability and preventing a loss of the information in high density storage devices. The magnetocrystalline anisotropy (MCA) shows very complex behavior depending on several factors such as thickness of films, interface structure, lattice distortion, and surface geometry^13^,^15^,^22^,^23^,^24^,^25^,^26^,^27^. In particular, the MCA energy is tunned by interface structures and epitaxial strain in multilayer systems^22^,^23^, which can be understood by modification of electronic structure based on perturbation theory^28^. Indeed, the L1_0_-MnGa alloy, which has equal chemical ratio of Mn and Ga, is known as FM materials with high PMA estimated as 10–15 Merg/cm^3 ^22,29,30. The PMA is observed in bilayer systems consisted with MnGa and Co, Fe and FeCo^1^,^2^,^3^.

In this paper, we investigate the magnetic interaction between L1_0_-MnGa and face-centered cubic (fcc) Co films depending on interface structure and lateral lattice constant (*a*). The enhanced magneto-crystalline anisotropy (MCA) driven by lattice expansion is discussed with magnetization reversal and interface interaction.

## Results and Discussion

**Figure 1:**
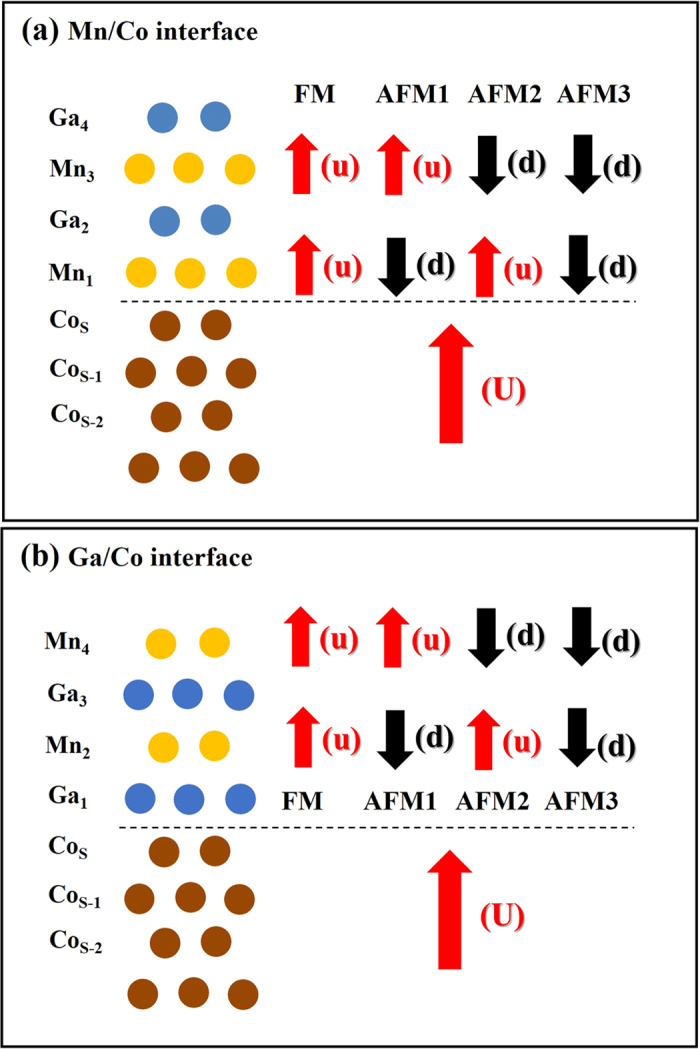
The schematic illustrations of the calculated structures with (**a**) Co/Mn and (**b**) Ga/Mn interface. Initial magnetic configurations are represented by red and back arrows, which are parallel and anti-parallel to the magnetization direction of Co layers, respectively. The initial magnetic states are only considered for the Mn and Co atoms. See text for more details.

In MnGa/Co(001) film calculations, two interfaces are considered due to polar surface of L1_0_-MnGa, Mn or Ga terminated surfaces. To find reasonable interface structure of Mn/Co or Ga/Co, we perform total energy calculation with four initial magnetic states. In Table 1, we show the calculated total energy differences according to the interface and initial magnetic configurations. As shown in Fig. 1, FM, AFM1, AFM2, and AFM3 correspond to uu/U, ud/U, du/U, and dd/U configuration, respectively. The magnetic states of Co layers are fixed to U, and u stands for the parallel spin direction of Mn atoms, and d for the anti-parallel spin configuration respect to Co layers. The first u is the spin configuration of Mn_3_ (Mn_4_) in Mn/Co (Ga/Co) interface and second one indicates spin configuration of Mn_1_ (Mn_2_), respectively. For instance, AFM3 (dd/U) means that the magnetization direction of MnGa layer is totally opposite to Co layers. The total energies of FM states with Mn/Co interface are set to be zero in total energy calculations for each *a* values. In Table 1, thus, the energy difference is calculated as 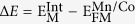, where M refers to the magnetic configurations (FM, AFM1, AFM2, or AFM3), and “Int” to Mn/Co or Ga/Co. Accordingly, systems with positive energy difference in Table 1 are less stable than FM-Mn/Co structure.

It is found the stable interface is independent on *a*, and the Mn/Co interface is energetically more favorable than Ga/Co interface. Interestingly, the magnetic interactions between L1_0_-MnGa and Co are changed from AFM3 to FM depending on *a* in Mn/Co interface. In contrast, magnetization reversal is not observed in Ga/Co interface. This clearly indicates that the FM ordering between Mn and Co stems from the change of the Mn-Co hybridization, rather than the lattice expansion. Recently, the anti-parallel magnetic coupling between Mn-Ga alloys and Co has been observed at *a* = 2.880 Å, and they have assumed that Mn-Ga/Co films dominantly form the Ga/Co interface, due to similar composition dependent magnetic behaviors of Mn-Ga/Co films comparing with Mn-Ga alloys^2^. Our computational result of Ga/Co interface well describes the experiment. Indeed, if both interfaces are thermally stable, the interface could be adjusted by growing modes, such as Co on MnGa or MnGa on Co. Thus, the control of interface is very important tunning the magnetic properties of MnGa/Co films. In the following, we discuss the Mn/Co interfacial systems, since they are energetically stable and show interesting magnetic behavior.

**Figure 2:**
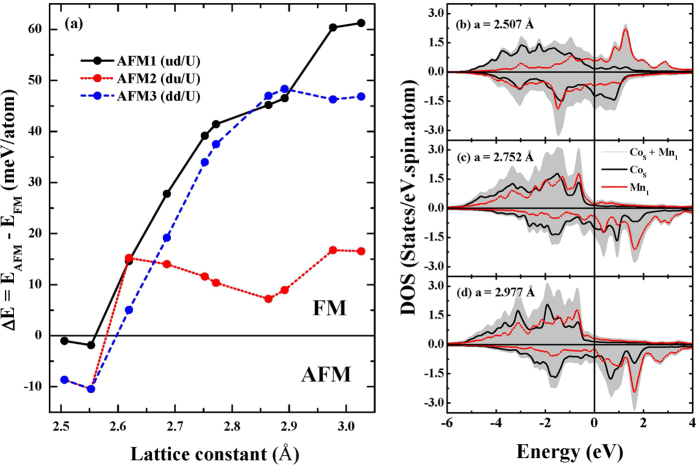
(**a**) Total energy difference between FM and AFM states as a function of lattice parameter (*a*). The DOSs of Co_*S*_ and Mn_1_ are presented for *a* (**b**) 2.507 Å, (**c**) 2.752 Å and (**c**) 2.977 Å. Calculations of the total energy difference and the DOS spectra are performed for Co/Mn interface.

To further confirm the magnetization reversal as a function of *a*, we calculate total energy difference between FM and AFM states, denoted as ΔE = E_AFM_ − E_FM_ in Fig. 2(a). The positive (negative) energy differences mean FM (AFM)ground state. One can see the AFM states at *a* = 2.507 and 2.553 Å, and the FM state appears from *a* = 2.618 Å. This result demonstrates that the epitaxial strain is an efficient way to tailor the magnetic interactions at the Mn/Co interfaces. We expect that in practice *a* can be adjusted by selection of substrates supporting the MnGa/Co films.

In Table 2, we display the calculated magnetic moment of Ga, Mn, and Co atoms within the muffin-tin (MT) sphere. For the AFM states, the magnetic moments of Co_*S*_ and Mn_1_ are reduced compared to those of Co_*S*−1_ and Mn_3_. For the FM states, however, the magnetic moments of Co_*S*_ and Mn_1_ are close to those of Co_*S*−1_ and Mn_3_, respectively. The suppressed magnetic moments can be understood by hybridization with neighboring layer. In AFM coupling, Mn_1_ induces electrons in minority spin states for Co_*S*_ because Mn_*S*_ has negative magnetic moment. Inversely, Co_*S*_ having positive magnetic moment provokes an increment of electrons in majority spin states for Mn_1_. Thus, the bilateral process between Mn_1_ and Co_*S*_ will decrease the spin asymmetry of the total number of electrons, which is also found in the spin polarized density of state spectra. In addition, one can see an enhancement of magnetic moments in Mn_1_ and Co_*S*_ depending on *a* (where Mn_1_ and Co_*S*_ stand for the atoms at the interface), and significant modifications are observed when the magnetic state changes from AFM to FM. It means that the spin asymmetry between majority and minority spin states is induced by epitaxial strain. The magnitude of magnetic moment is simply obtained by the difference of the electrons in the two spin parts, majority and minority.

In Fig. 2(b–d), we present the electronic density of states (DOS) of Mn_1_ and Co_*S*_ atoms at *a* = 2.507 Å (AFM), 2.752 Å (FM) and 2.977 Å (FM) to monitor the magnetic behavior from electronic structure. In AFM state, the shape of DOS spectra of Co_*S*_ and Mn_1_ are broad. In addition, hybridization between Co_*S*_ and Mn_1_ is observed in large range of minority spin state. In FM states with *a* = 2.752 and 2.977 Å, marked differences are seen in the DOS compared to the AFM state. One can observe the DOS reversals of spin states. This phenomenon correlates well with the magnetization reversal. In addition, a weak hybridization is obtained with larger in-plane lattice constant. Interestingly, more peaks are observed in majority spin part when *a* is expanded. This indicates that Co_*S*_ and Mn_1_ become more localized with increasing *a*. Furthermore, the DOS of both Co_*S*_ and Mn_1_ near E_F_ is decreased with increasing *a*. It indicates electron loss in the MT region and charge redistribution between MT and interstitial region. When *a* is expanded, the inter-atomic distance between Mn_1_ and Co_*S*_ increases corresponding to longer bond length. As a result, the charge redistribution is essential to maintain Co-Co, Mn-Mn and Co-Mn bonding.

**Figure 3:**
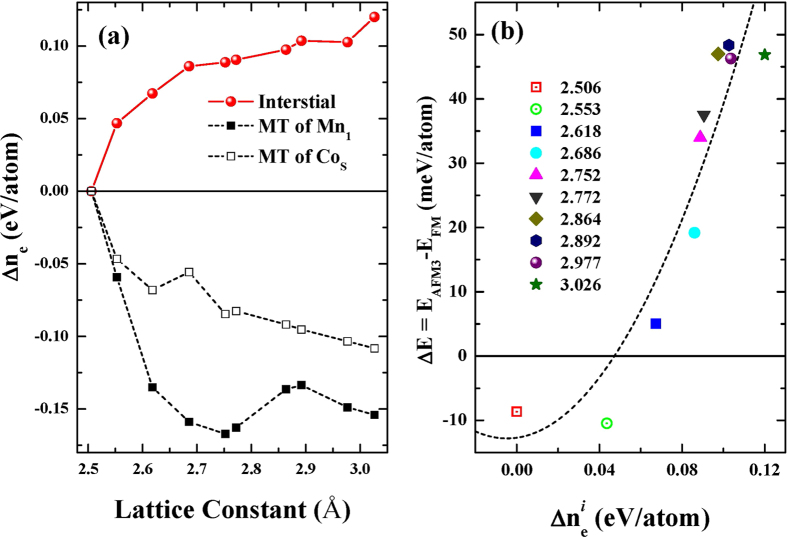
(**a**) The charge differences for MT (Mn_1_ and Co_*S*_) and interstitial region plotted as a function of lattice constant. (**b**) Energy different as a function of interstitial charge difference. The dashed line stands for the second order fitting.

According to Heilter and London (HL) model for magnetic ordering of H_2_, weaker hybridization and more localized electron prefer the FM order resulting in a gain in the magnetic energy^17^,^18^. As suggested above, the weaker hybridization and localized effect originates from charge redistribution. In Fig. 3(a), we display the relative number of electrons (Δ*n*_*e*_) as a function of *a*, with respect to those at *a* = 2.507 Å system. Indeed, it is clearly observed that a loss of electrons from the MT sphere of Mn_1_ and Co_*S*_, and a gain of electrons in the interstitial region with increasing *a*. The loss of electrons mainly occurs in specific spin part to increase magnitude of magnetic moment of each atoms (not shown here), and this is confirmed from enhancement of magnetic moment with increasing *a* in Table 2. Therefore, the increased spin asymmetry is mainly originated from the charge redistribution which can be simply parameterized by charge difference in interstitial region 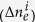. The increased spin asymmetry between Mn_1_ and Co_*S*_ may affect the magnetic ordering between them. The magnetic ordering can be determined by competition between Coulomb and kinetic energies.

According to Stoner model, total energy variation due to electron transfer is expressed with kinetic and magnetic energies^31^. The magnetic energy is proportional to square of charge and spin asymmetry, and kinetic energy is linear function of charge and spin asymmetry. The charge redistribution and the modified spin asymmetry can induce changes in the magnetic order. Therefore, we should be able to represent the magnetization reversal of MnGa/Co films in terms of 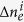, which is a parameter including the charge redistribution and the change of the spin asymmetry.

In Fig. 3(b), we plot the energy difference between FM and AFM3 as a function of 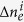. The Δ*E* is fitted with a quadric function where the coefficients may be interpreted as the Coulomb (Δ*E*_*M*_) and kinetic (Δ*E*_*K*_) energies, viz,


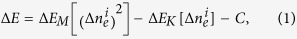


where C is a positive constant. We notice that whereas the data in Fig. 3(b) weakly dependence on the muffin-tin radius as going from 2.15/2.25 to 2.25/2.35 a.u. for the 3d transition metals/Ga atoms (shown are results only for radii 2.20/2.30 a.u.), the conclusions below are not affected by the actual MT radius. According to Fig. 3(b), the magnetic energy difference can be understood as the competition between the Coulomb and the kinetic energies. For AFM states which have negative Δ*E* and small charge redistribution, the kinetic energy terms should have larger contribution. On the other hand, the FM states corresponding to positive energy difference dominantly have Coulomb interaction terms. It is concluded that from the combination of the HL and Stoner model the weak hybridization and localized states due to charge redistribution may induce modification of magnetic interactions and this well describes the predicted magnetization reversal as a function of *a*.

**Figure 4:**
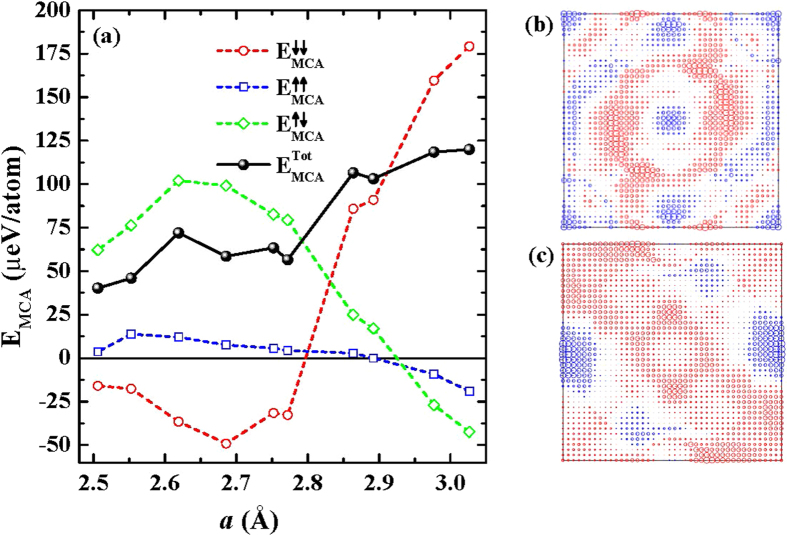
(**a**) Calculated MCA energies of the total (black filled circles), minority-minority (red open circles), majority-majority (blue open squares) and majority-minority (green open diamonds) spin-orbit channels, are displayed. Distributions of MCA energies over two-dimensional BZ at *a* of (**b**) 2.507 Å and (**c**) 2.977 Å. See main text for explanation.

Next we discuss the MCA energies as a function of *a*. As shown above, interface and in-plane lattice constant are essential factors for tailoring the magnetic structure of MnGa/Co films. Previously, we found that the MCA energy (E_MCA_) of L1_0_ MnGa alloy can be tuned by epitaxial strain^22^. Here, we calculate the E_MCA_ (in *μ*eV/atom, including Co, Mn and Ga atoms) depending on *a* using the torque method^32^. In film structure, the E_MCA_, arising from spin-orbit coupling (SOC), is written as 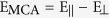, where 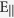 and 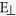 correspond total energies with in-plane and perpendicular magnetization to film surface, respectively. Therefore, positive MCA energies are associated with PMA, and negative ones correspond to in-plane magnetization. In Fig. 4(a), we display the calculated E_MCA_. All MnGa/Co films show large PMA, and E_MCA_ is increased with *a*. It seems like that the enhanced MCA energy results mainly from the epitaxial strain effect on MnGa alloys. However, the interface effects cannot be ignored due to the *a*-dependent hybridization between Mn_1_ and Co_*S*_ layers. To check the interface effect on MCA energy, we also calculate E_MCA_ for the Ga/Co interface with *a* = 2.507 Å and 2.752 Å. The so obtained MCA energies, 59.46 *μ*eV/atom and 28.69 *μ*eV/atom, respectively, show opposite trend compare to the Mn/Co interface. This means that the interaction at the interface is important to understand the *a*-dependent MCA energy.

To reveal the origin of the PMA and enhancement of the MCA energy with increasing *a*, we explore the distribution of the E_MCA_ over two-dimensional (2D) Brillouin Zone (BZ) as shown in Fig. 4(b,c). The circles are contributions of spin-orbit interaction between the occupied and unoccupied state at given *k*-points. Red (blue) circles mean perpendicular (in-plane) magnetization. The magnitude of E_MCA_ is proportional to the size of circles. Thus, total E_MCA_ is determined by sum of E_MCA_ at given *k*-points over 2D-BZ. One can see that there is no dominant PMA contributions of single point nor any particular directions. Furthermore, the changes of E_MCA_ and direction of magnetization occur around not only zone center (Γ) but also around corners (M). Actually, the modification of MAE with increasing *a* is observed in the whole *k*-space. We think that no simple picture can explain the PMA and magnetic anisotropy behavior as a function of *a*.

According to perturbation theory^28^, E_MCA_ is defined by the SOC interaction between occupied and unoccupied states with magnetic quantum number (*m*) through the *l*_*z*_ and *l*_*x*_ operators, as


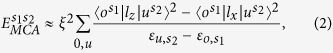


where *o* (*u*) and 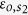 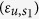 represent eigenstates and eigenvalues of occupied (unoccupied), respectively. *s*_1_ (*s*_2_) is spin state of occupied (unoccupied) states, majority(↑) or minority(↓) spin, and *ξ* means the SOC strength. From Eq. (2), the MCA can be analyzed by decomposing E_MCA_ into spin channels, namely 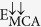, 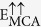, and 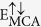. For the same spin channel interaction, the positive contribution to E_MCA_ comes from the SOC between occupied and unoccupied state with the same *m* through the *l*_*z*_ operator. On the other hand, the SOC with the different *m* through the *l*_*z*_ operator has positive contribution for spin-flip channel interaction^15^,^23^,^24^.

**Figure 5:**
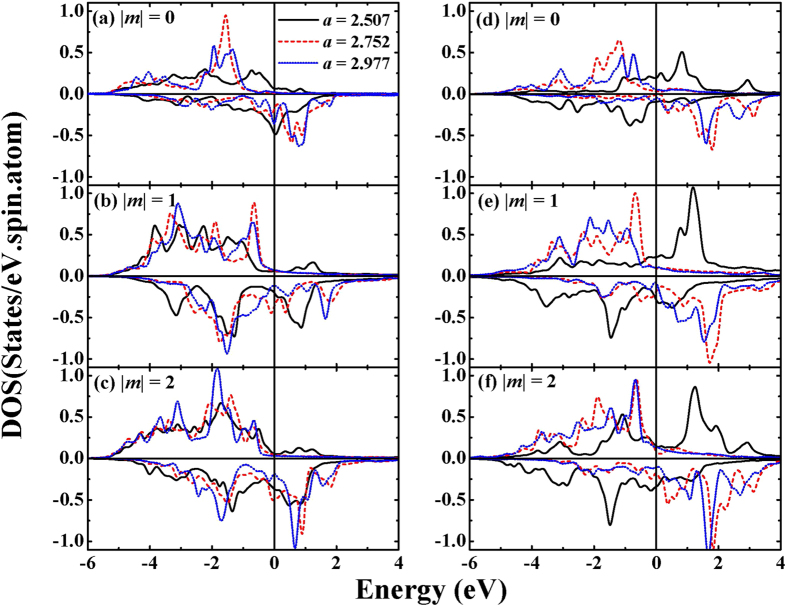
m-resolved DOS spectra of (**a–c**) Co_*S*_ and (**d–f**) Mn_1_ atoms are displayed. The systems with *a* = 2.507 Å, 2.752 Å, and 2.977 Å are plotted by black solid, red dashed, and blue dotted lines, respectively. The magnetic quantum numbers are shown in each panels.

In Fig. 4(a), it is observed that the major contribution of SOC channel to the total E_MCA_ is changed from 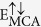 to 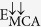 with increasing *a*. Thus, the changes of E_MCA_ are understood by modifications of the dominant E_MCA_ contributions. The MCA behavior can also be analyzed by the *m*-resolved DOS (*m*-DOS) of *d* electrons, shown in Fig. 5. For *a* = 2.507 Å, the dominant SOC channel is spin-flip, 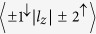 and 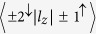 from Co_*S*_ to Mn_1_, because of less unoccupied majority spin states of Co_*S*_ and minority spin state of Mn_1_. For FM with *a* = 2.752 Å, PMA mainly originates from spin-flip interaction similarly to AFM (*a* = 2.507 Å), but the electronic origin is 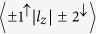 from Co_*S*_ to Mn_1_ or Mn_1_ to Co_*S*_. These changes should be ascribed to the magnetization reversal. At large *a* (*a* = 2.977 Å), the dominant contribution of SOC channel is clearly 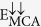. One can see the significantly increased DOS (minority spin states) near E_F_ with 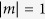 (Co_*S*_ and Mn_1_) and 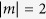 (Co_*S*_). Therefore, it can be inferred that the 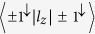 between Co_*S*_ and Mn_1_, and 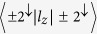 between Co_*S*_, lead to the PMA. The latter can be understood by weaker hybridization and localized effects. This reflects the important role of interface interaction for MAE. Hence, we suggest that both interfacial interactions and magnetization reversal are important factors to explain the enhancement of E_MCA_.

## Conclusion

In summary, we have investigated magnetic properties of *L*1_0_-MnGa on fcc Co (001) film depending on interface structure and in-plane lattice constant. We have obtained magnetization reversal from AFM to FM coupling between *L*1_0_-MnGa and fcc Co (001) layers as a function of *a*. In Mn/Co interface structures, the reason for the *a*-dependent magnetization reversal is found to be the weak hybridization and more localized electrons due to charge re-distribution between MT and interstitial region. Furthermore, all MnGa/Co(001) films show perpendicular magnetic energy, and the magnetocrystalline anisotropy energy is enhanced with increasing *a*. The behavior of magnetic anisotropy energy can be explained by interface interaction and magnetization reversal. Finally, we have realized that the magnetic properties of MnGa/Co film can be tailored by controlling of the interface interaction, and the change of the in-plane lattice constant is one of the most effective methods.

## Methods

We have employed the thin film version of all-electron full potential linearized augmented plane (FLAPW) method. Therefore, no shape approximation is assumed in charge, potential, and wave-function expansions^33^,^34^,^35^. We treat the core electrons fully relativistically, and the spin orbit interaction among valence electrons are dealt with second variationally^36^. The generalized gradient approximation (GGA) exchange-correlation potentials is used to describe exchange and correlation interaction^37^. Spherical harmonics with *l*_*max*_ = 8 are used to expand the charge, potential, and wave-functions in the muffin tin region. Energy cut-offs of 225 Ry and 13.7 Ry are implemented for the plane wave star function and basis expansions in the interstitial region. We use 21 × 21 *k*-points with the Monkhorst-Pack method^38^. The muffin-tin radius is considered as 2.2 a.u. for 3d transition metals and 2.3 a.u. for Ga atom. The muffin-tin radii for all atoms are kept constant upon lateral lattice constant change. We assume four layers of *L*1_0_-MnGa film, consisting of two Mn and two Ga atoms grown pesudomorphically on fcc Co p(1 × 1) sublayer. The Co sublayer is simulated by seven fcc Co(001) layers. To apply epitaxial strain, we change *a* of the film. Here, we select *a* values corresponding to certain substrates, such as Co, Cu, Pd, Pt, Al, MgO and InAs. The vertical distance of films with various *a* is fully relaxed with force and total energy minimization procedure. The convergence for all physical quantities investigated in the present work has been carefully checked. The Co atom at the interface between MnGa and fcc Co (001) surface is denoted by Co_*S*_ and the subsurface layers by Co_*s*−*i*_. Furthermore, Mn_*i*_ means the i-th ad-layer counted from interface (Fig. 1).

## Additional Information

**How to cite this article**: Kim, D. and Vitos, L. Tuned Magnetic Properties of L1_0_-MnGa/Co(001) Films by Epitaxial Strain. *Sci. Rep.* **6**, 19508; doi: 10.1038/srep19508 (2016).

## Figures and Tables

**Table 1:** Calculated total energy different (in meV/atom) according to interface structures and initial magnetic states with *a* = 2.507 and 2.752 Å.

Interface (*a*)	FM	AFM1	AFM2	AFM3
Mn/Co (2.506)	0.0	−1.03	AFM3	−8.64
Ga/Co (2.506)	78.34	95.67	89.25	77.06
Mn/Co (2.752)	0.0	39.2	11.6	34.0
Ga/Co (2.752)	71.6	77.8	75.3	70.5

Negative values indicate stable states relative to the FM state of the Mn/Co interface. The AFM3 in the column of AFM2 means that the initial AFM2 state is changed to AFM3 after self-consistent calculation.

**Table 2:** Calculated spin magnetic moment of Ga, Mn and Co atoms in MT sphere.

*a*	2.506	2.553	2.618	2.752	2.977	3.026
Ga_4_	−0.015	−0.011	0.008	0.014	0.028	0.020
Mn_3_	−2.467	−2.524	2.521	2.600	3.082	3.085
Ga_2_	0.059	0.069	−0.096	−0.096	−0.087	−0.091
Mn_1_	−1.504	−1.725	2.715	2.921	2.962	2.974
Co_*S*_	1.255	1.312	1.630	1.724	1.727	1.696
Co_*S*−1_	1.706	1.739	1.774	1.838	1.802	1.810

The presented values are magnetic moments for Mn/Co interface structures.
